# Supplementary material for: Development and Validation of Case-Finding Algorithms for Digestive Cancer in the Spanish Healthcare Database BIFAP
Source: J Clin Med. 2024 Jan 9;13(2):361. doi: 10.3390/jcm13020361 (PMC10816118; doi:10.3390/jcm13020361)
Supplement: Supplementary file 1 [file jcm-13-00361-s001.zip › jcm-2795979-supplementary.pdf]

SUPPLEMENTARY MATERIAL

Development and Validation of Case-Finding Algorithms for Digestive Cancer in the Spanish Healthcare Database BIFAP

Encarnación Fernández-Antón, Antonio Rodríguez-Miguel, Miguel Gil, Amelia Castellano-López and Francisco J. de Abajo

Supplementary Methods 1: BIFAP digestive cancer case-finding algorithms.

ESOPHAGEAL CANCER

Case-finding algorithm for electronic medical records software using as reference:

1) ICPC-BIFAP codes

ICPC code	BIFAP subcode	Description
D77	2	Esophageal Cancer (Malignant neoplasm)
D77	8	Esophageal Adenocarcinoma

2) Text mining search strategies

Number	Concept	Specific strings
[1]	String text location	ESOFAGO ***OR*** ESOFAGICO
[2]	String text malignant cancer	ADENOCARCINOMA ***OR*** CANCER ***OR*** CARCINOMA ***OR*** (CA ***AND NOT*** BENIGNO) ***OR*** (NEOPLASIA ***AND NOT*** BENIGNO) ***OR*** (NEO ***AND NOT*** BENIGNO) ***OR*** TM ***OR*** "TUMOR MALIG"
[3]	Other string text suggesting malignancy	METASTASIS ***OR*** MTX ***OR*** MTXS ***OR*** METAS ***OR*** MET ***OR*** METX ***OR*** METXS ***OR*** MTTS ***OR*** PALIATIVOS ***OR*** QUIMIOTERAPIA ***OR*** RADIOTERAPIA ***OR*** T1N% ***OR*** T2N% ***OR*** T3N% ***OR*** T4N%

Case-finding Algorithm syntax: [1] AND ([2] OR [3])

3) ICD-9 codes

ICD9 code	Description
150	Malignant neoplasm of esophagus
150.0	Malignant neoplasm of cervical esophagus
150.1	Malignant neoplasm of thoracic esophagus
150.2	Malignant neoplasm of abdominal esophagus
150.3	Malignant neoplasm of upper third of esophagus

150.4	Malignant neoplasm of middle third of esophagus
150.5	Malignant neoplasm of lower third of esophagus
150.9	Malignant neoplasm of esophagus, unspecified site
230.1	Carcinoma in situ of esophagus

GASTRIC CANCER

Case-finding algorithm for electronic medical records software using as reference:

1) <u>ICPC-BIFAP codes</u>

ICPC code	BIFAP subcode	Description
D74	1	Gastric Cancer (Malignant neoplasm)
D75	1003	Gastric Adenocarcinoma

2) <u>Text mining search strategies</u>

Number	Concept	Specific strings
[1]	String text location	ESTOMAGO ***OR*** GASTRIC ***OR*** CARDIAS ***OR*** PILORO
[2]	String text malignant cancer	ADENOCARCINOMA ***OR*** CANCER ***OR*** CARCINOMA ***OR*** (CA ***AND NOT*** BENIGNO) ***OR*** (NEOPLASIA ***AND NOT*** BENIGNO) ***OR*** (NEO ***AND NOT*** BENIGNO) ***OR*** TM ***OR*** "TUMOR MALIG"
[3]	Other string text suggesting malignancy	METASTASIS ***OR*** MTX ***OR*** MTXS ***OR*** METAS ***OR*** MET ***OR*** METX ***OR*** METXS ***OR*** MTTS ***OR*** PALIATIVOS ***OR*** QUIMIOTERAPIA ***OR*** RADIOTERAPIA ***OR*** T1N% ***OR*** T2N% ***OR*** T3N% ***OR*** T4N%

Case-finding Algorithm syntax: [1] AND ([2] OR [3])

3) <u>ICD-9 codes</u>

ICD9 code	Description
151	Malignant neoplasm of stomach
151.0	Malignant neoplasm of cardia
151.1	Malignant neoplasm of pylorus
151.2	Malignant neoplasm of pyloric antrum
151.3	Malignant neoplasm of fundus of stomach
151.4	Malignant neoplasm of body of stomach
151.5	Malignant neoplasm of lesser curvature of stomach, unspecified
151.6	Malignant neoplasm of greater curvature of stomach, unspecified
151.8	Malignant neoplasm of other specified sites of stomach
151.9	Malignant neoplasm of stomach, unspecified site

230.2	Carcinoma in situ of stomach

PANCREATIC CANCER

Case-finding algorithm for electronic medical records software using as reference:

1) ICPC-BIFAP codes

ICPC code	BIFAP subcode	Description
D76	1	Pancreatic Cancer (Malignant neoplasm)
D76	4	Pancreatic Carcinoma
D76	5	Pancreatic Adenocarcinoma

2) Text mining search strategies

Number	Concept	Specific strings
[1]	String text location	PANCRE
[2]	String text malignant cancer	ADENOCARCINOMA ***OR*** CANCER ***OR*** CARCINOMA ***OR*** (CA ***AND NOT*** BENIGNO) ***OR*** (NEOPLASIA ***AND NOT*** BENIGNO) ***OR*** (NEO ***AND NOT*** BENIGNO) ***OR*** TM ***OR*** “TUMOR MALIG”
[3]	Other string text suggesting malignancy	METASTASIS ***OR*** MTX ***OR*** MTXS ***OR*** METAS ***OR*** MET ***OR*** METX ***OR*** METXS ***OR*** MTTS ***OR*** PALIATIVOS ***OR*** QUIMIOTERAPIA ***OR*** RADIOTERAPIA ***OR*** T1N% ***OR*** T2N% ***OR*** T3N% ***OR*** T4N%

Case-finding Algorithm syntax: [1] AND ([2] OR [3])

3) ICD-9 codes

ICD9 code	Description
157	Malignant neoplasm of pancreas
157.0	Malignant neoplasm of head of pancreas
157.1	Malignant neoplasm of body of pancreas
157.2	Malignant neoplasm of tail of pancreas
157.3	Malignant neoplasm of pancreatic duct
157.4	Malignant neoplasm of islets of langerhans
157.8	Malignant neoplasm of other specified sites of pancreas
157.9	Malignant neoplasm of pancreas, part unspecified
M8154/3	Mixed Adenocarcinoma of insular and exocrine cells

HEPATOBILIARY CANCER

Case-finding algorithm for electronic medical records software using as reference:

1) ICPC-BIFAP codes

ICPC code	BIFAP subcode	Description
D77	4	Liver Cancer, hepatocarcinoma (Malignant neoplasm)
D77	5	Gallbladder Cancer

2) Text mining search strategies

Number	Concept	Specific strings
[1]	String text location	HIGADO ***OR*** HEPAT ***OR*** BILIAR ***OR*** VESICULA ***OR*** AMPULOMA ***OR*** COLANGIOCARC ***OR*** HEPATOCA ***OR*** HEPATOBLASTOM
[2]	String text malignant cancer	ADENOCARCINOMA ***OR*** CANCER ***OR*** CARCINOMA ***OR*** (CA ***AND NOT*** BENIGNO) ***OR*** (NEOPLASIA ***AND NOT*** BENIGNO) ***OR*** (NEO ***AND NOT*** BENIGNO) ***OR*** TM ***OR*** "TUMOR MALIG"
[3]	Other string text suggesting malignancy	METASTASIS ***OR*** MTX ***OR*** MTXS ***OR*** METAS ***OR*** MET ***OR*** METX ***OR*** METXS ***OR*** MTTS ***OR*** PALIATIVOS ***OR*** QUIMIOTERAPIA ***OR*** RADIOTERAPIA ***OR*** T1N% ***OR*** T2N% ***OR*** T3N% ***OR*** T4N%

Case-finding Algorithm syntax: [1] AND ([2] OR [3])

3) ICD-9 codes

ICD9 code	Description
155	Malignant neoplasm of liver and intrahepatic bile ducts
155.0	Malignant neoplasm of liver, primary
155.1	Malignant neoplasm of intrahepatic bile ducts
155.2	Malignant neoplasm of liver, not specified as primary or secondary
156	Malignant neoplasm of gallbladder and extrahepatic bile ducts
156.0	Malignant neoplasm of gallbladder
156.1	Malignant neoplasm of extrahepatic bile ducts
156.2	Malignant neoplasm of ampulla of vater
156.8	Malignant neoplasm of other specified sites of gallbladder and extrahepatic bile ducts
156.9	Malignant neoplasm of biliary tract, part unspecified site
230.8	Carcinoma in situ of liver and biliary system
M8160/3	Cholangiocarcinoma

M8161/3	Cystadenocarcinoma Of Biliary Tract
M8170/3	Hepatocellular Carcinoma
M8180/3	Combined Cholangio and Hepatocellular Carcinoma
M8970/3	Hepatoblastoma
M8970/6	Metastatic Hepatoblastoma

COLORECTAL CANCER

Case-finding algorithm for electronic medical records software using as reference:

1) ICPC-BIFAP codes

ICPC code	BIFAP subcode	Description
D75	1	Colon Cancer (Malignant neoplasm)
D75	4	Rectum Cancer
D75	5	Colorectal Cancer
D75	6	Sigma Cancer
D75	999	Colon Adenocarcinoma
D75	1004	Sigma Adenocarcinoma
D75	1005	Rectum Adenocarcinoma

2) Text mining search strategies

Number	Concept	Specific strings
[1]	String text location	RECTO ***OR*** RECTAL ***OR*** SIGMA **OR** SIGMOID ***OR*** COLON ***OR*** CIEGO ***OR*** COLORECTAL ***OR*** CECAL ***OR*** CECAL **OR** "INTESTINO GRUESO" ***OR*** RECTOSIGMA
[2]	String text malignant cancer	ADENOCARCINOMA ***OR*** CANCER ***OR*** CARCINOMA ***OR*** (CA ***AND NOT*** BENIGNO) ***OR*** (NEOPLASIA ***AND NOT*** BENIGNO) ***OR*** (NEO ***AND NOT*** BENIGNO) ***OR*** TM ***OR*** "TUMOR MALIG"
[3]	Other string text suggesting malignancy	METASTASIS ***OR*** MTX ***OR*** MTXS ***OR*** METAS ***OR*** MET ***OR*** METX ***OR*** METXS ***OR*** MTTS ***OR*** PALIATIVOS ***OR*** QUIMIOTERAPIA ***OR*** RADIOTERAPIA ***OR*** T1N% ***OR*** T2N% ***OR*** T3N% ***OR*** T4N%
[4]	Fine-tuning string text location	(ANGUL ***AND*** HEPAT) ***OR*** (ANGUL ***AND*** ESPLEN)

Case-finding Algorithm syntax: [1] AND ([2] OR [3])

3) ICD-9 codes

ICD9 code	Description
153	Malignant neoplasm of colon
153.0	Malignant neoplasm of hepatic flexure
153.1	Malignant neoplasm of transverse colon
153.2	Malignant neoplasm of descending colon
153.3	Malignant neoplasm of sigmoid colon
153.4	Malignant neoplasm of cecum
153.6	Malignant neoplasm of ascending colon
153.7	Malignant neoplasm of splenic flexure
153.8	Malignant neoplasm of other specified sites of large intestine
153.9	Malignant neoplasm of colon, unspecified site
154	Malignant neoplasm of rectum rectosigmoid junction and anus
154.0	Malignant neoplasm of rectosigmoid junction
154.1	Malignant neoplasm of rectum
154.8	Malignant neoplasm of other sites of rectum, rectosigmoid junction and anus
230.3	Carcinoma in situ of colon
230.4	Carcinoma in situ of rectum

Other considerations:

- Cases identified with the previous text mining strategy are removed if there are keywords in the vicinity related to screening, family (other relative's diseases), speculation (??) or negation.

Supplementary Methods 2: Hierarchical searching of the case-finding algorithms

Cancer group	Order
Malignant neoplasm of esophagus	1
Malignant neoplasm of pancreas	1
Malignant neoplasm of colorectum	1
Malignant neoplasm of stomach	2
Malignant neoplasm of hepatobiliary tract	3
Other malignant neoplasm of digestive tract	4
Rest of malignant neoplasm	5
Metastasis	6

Case-finding algorithms were hierarchically structured. When two diagnoses coexist, they were labelled based on order priority. For instance, a "rectal cancer with liver metastasis" could be detected by the case-finding algorithms as “malignant neoplasm of colorectum” and as “malignant neoplasm of hepatobiliary tract cancer”, but finally labelled as "Malignant neoplasm of colorectum" as it has higher priority (order 1) than "Malignant neoplasm of hepatobiliary tract" which is order 3.

Supplementary Table S1: Validation results before and after fine-tuning.

Type of cancer	Before fine-tuning PPV (95%CI), %	After fine-tuning PPV (95%CI), %
Hepato-biliary (n=124)	71.1 (63.5-78.7)	87.6 (81.8-93.4)
Esophageal (n=152)	92.8 (88.7-96.9)	96.2 (93.1-99.2)
Pancreatic (n=152)	89.8 (84.9-94.7)	89.4 (84.5-94.3)
Gastric (n=158)	87.9 (82.7-93.0)	92.5 (88.3-96.6)
Colorectal (n=174)	94.7 (91.2-98.2)	95.2 (92.1-98.4)
Overall (n=760)	90.5 (88.5-92.4)	92.4 (90.5-94.3)

Supplementary Table S2: Sensitivity analysis

		UNSUPPORTED CASES AS **VALID**	UNSUPPORTED CASES AS **NON VALID**	**EXCLUDING** UNSUPPORTED CASES
**Type of cancer**	**Information category**	**PPV (95CI%)**	**PPV (95CI%)**	**PPV (95CI%)**
Overall n=816 (56 unsupported cases)	1) Cancer diagnosis+ Clinical notes as free-text+ supporting information	92.8 (89.8-95.1)	92.5 (89.5-94.9)	93.0 (90.0-95.2)
	2) Cancer diagnosis+ Clinical notes as free-text	90.1 (85.2-93.9)	84.2 (78.5-89.0)	89.5 (84.3-93.5)
	3) Cancer diagnosis	93.5 (89.1-96.5)	72.4 (65.6-78.5)	91.7 (86.3-95.5)
	4) Weighted mean	92.6 (90.8-94.4)	90.4 (88.4-92.4)	92.4 (90.6-94.3)
Hepato-biliary n=143 (19 unsupported cases)	1) Cancer diagnosis+ Clinical notes as free-text+ supporting information	88.5 (77.8-95.3)	88.5 (77.8-95.3)	88.5 (77.8-95.3)
	2) Cancer diagnosis+ Clinical notes as free-text	87.8 (73.8-95.9)	80.5 (65.1-91.2)	86.8 (71.9-95.6)
	3) Cancer diagnosis	87.8 (73.8-95.9)	48.8 (32.9-64.9)	80.0 (59.3-93.2)
	4) Weighted mean	88.3 (83.1-93.6)	84.2 (78.2-90.2)	87.6 (81.8-93.4)
Esophageal n=159 (7 unsupported cases)	1) Cancer diagnosis+ Clinical notes as free-text+ supporting information	96.4 (89.9-99.3)	96.4 (89.9-99.3)	96.4 (89.9-99.3)
	2) Cancer diagnosis+ Clinical notes as free-text	92.3 (79.1-98.3)	87.2 (72.6-95.7)	91.9 (78.1-98.3)
	3) Cancer diagnosis	100	86.1 (70.5-95.3)	100
	4) Weighted mean	96.2 (93.3-99.2)	94.9 (91.5-98.3)	96.2 (93.1-99.2)
Pancreatic n=166 (14 unsupported cases)	1) Cancer diagnosis+ Clinical notes as free-text+ supporting information	88.2 (79.4-94.2)	88.2 (79.4-94.2)	88.2 (79.4-94.2)
	2) Cancer diagnosis+ Clinical notes as free-text	95.1 (83.4-99.4)	87.8 (73.8-95.9)	94.7 (82.3-99.4)
	3) Cancer diagnosis	92.5 (19.6-98.4)	65.0 (48.3-79.4)	89.7 (72.6-97.8)
	4) Weighted mean	89.6 (85.0-94.3)	86.5 (81.3-91.7)	89.4 (84.5-94.3)
Gastric n=165 (7 unsupported cases)	1) Cancer diagnosis+ Clinical notes as free-text+ supporting information	94.0 (86.5-98.0)	94.0 (86.5-98.0)	94.0 (86.5-98.0)
	2) Cancer diagnosis+ Clinical notes as free-text	81.0 (65.9-91.4)	76.2 (60.5-87.9)	80.0 (64.4-90.9)
	3) Cancer diagnosis	95.0 (83.1-99.4)	82.5 (67.2-92.7)	94.3 (80.8-99.3)
	4) Weighted mean	92.6 (88.6-96.6)	91.3 (87.0-95.6)	92.5 (88.3-96.6)
Colorectal n=183 (9 unsupported cases)	1) Cancer diagnosis+ Clinical notes as free-text+ supporting information	96.0 (90.2-98.9)	94.1 (87.5-97.8)	96.0 (90.0-98.9)
	2) Cancer diagnosis+ Clinical notes as free-text	90.0 (76.3-97.2)	85.0 (70.2-94.3)	89.5 (75.2-97.1)
	3) Cancer diagnosis	92.9 (80.5-98.5)	81.0 (65.9-91.4)	91.9 (78.1-98.3)
	4) Weighted mean	95.4 (92.4-98.4)	92.5 (88.7-96.3)	95.2 (92.1-98.4)

Supplementary Table S3: Stratified PPVs by sex.

Type of cancer	Sex	Non-valid case	Valid case	Total	PPV (95CI%)
Hepato-biliary (n=124)	Men	12	74	86	86.7 (79.8-94.5)
	Women	5	33	38	86.3 (73.4-99.2)
Esophageal (n=152)	Men	5	134	139	96.1 (91.1-100.0)
	Women	1	12	13	90.0 (85.5-94.5)
Pancreatic (n=152)	Men	10	71	81	86.1 (77.4-94.8)
	Women	5	66	71	93.4 (86.1-100.0)
Gastric (n=158)	Men	8	92	100	94.5 (89.8-99.2)
	Women	7	51	58	88.4 (78.5-98.2)
Colorectal (n=174)	Men	6	114	120	96.3 (92.7-100.0)
	Women	5	49	54	92.3 (84.5-100.0)
Overall (n=760)	Men	38	486	524	93.2 (90.6-95.7)
	Women	24	212	236	91.2 (86.9-95.4)
